# High-throughput sequencing of *Strongyloides stercoralis* – a fatal disseminated infection in a dog

**DOI:** 10.1017/S0031182024000568

**Published:** 2024-05

**Authors:** Eva Nosková, Vlasta Svobodová, Vilma Hypská, Argiñe Cerezo-Echevarria, Terézia Kurucová, Vladislav Ilík, David Modrý, Barbora Pafčo

**Affiliations:** 1 Department of Botany and Zoology, Faculty of Science, Masaryk University, Kotlářská 2, 611 37 Brno, Czech Republic; 2 Institute of Vertebrate Biology, Czech Academy of Sciences, Květná 8, 603 00 Brno, Czech Republic; 3 Department of Pathology and Parasitology, University of Veterinary Sciences Brno, Palackého třída 1946/1, 612 42, Brno, Czech Republic; 4 Veterinary centrum VIVA, Medkova 6, 500 02 Hradec Králové, Czech Republic; 5 Pathology Department, LABOKLIN GmbH & Co. KG, 97688 Bad Kissingen, Germany; 6 Central European Institute of Technology, Masaryk University, Kamenice 753/5, 625 00 Brno, Czech Republic; 7 Department of Veterinary Sciences, Faculty of Agrobiology, Food and Natural Resources, Czech University of Life Sciences Prague, Kamýcká 129, 165 00 Prague, Czech Republic; 8 Institute of Parasitology, Biology Centre, Czech Academy of Sciences, Branišovská 31, 370 05 České Budějovice, Czech Republic

**Keywords:** dog, high-throughput sequencing, *Strongyloides stercoralis*, Strongyloidiasis, zoonotic

## Abstract

The rhabditid nematode *Strongyloides stercoralis* is known worldwide as the causative agent of strongyloidiasis in humans. In addition to public health concerns, *S*. *stercoralis* also infects dogs, which represent a possible reservoir for potentially zoonotic transmissions. We describe the first confirmed case of fatal disseminated infection in a dog in the Czech Republic. The microscopic and histological results were supported by a complex genotyping approach. Using high-throughput sequencing of the hypervariable region (HVR-IV) of 18S rDNA and Sanger sequencing of the partial cytochrome c oxidase subunit 1 gene (*cox1*), the potentially zoonotic haplotype/lineage A of *S*. *stercoralis* was confirmed, while the solely canine haplotype/lineage B was not found. The development of the disease is mainly associated with immunodeficiency, and in this case, it was triggered by inappropriate treatment, in particular the use of corticosteroids.

## Graphical abstract

**Figure:**
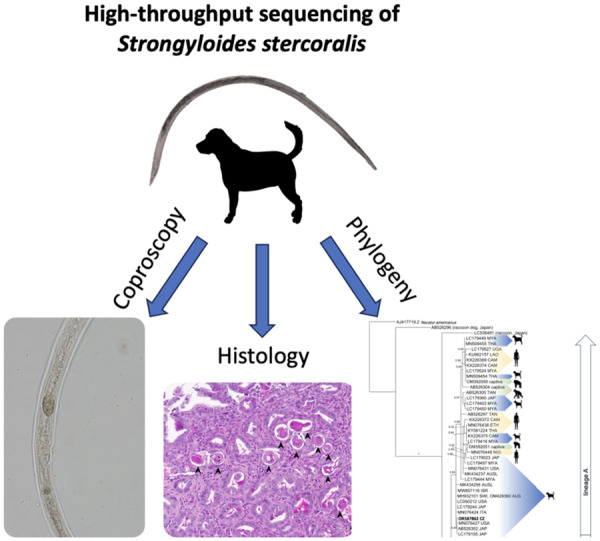

## Introduction

*Strongyloides stercoralis* infects a range of hosts, including canids, felids, humans and non-human primates (NHPs). Resulting infection, usually termed strongyloidiasis, remains a major veterinary and public health challenge globally (Bradbury *et al*., 2021). The parasite has a unique life cycle in which it alternates between free-living and parasitic stages. The free-living stage is a single generation with a short life span. The parasitic generations include only parthenogenetic females living in the small intestine, that are ovoviviparous. The autoinfective filariform larvae may develop from rhabditiform larvae already in the intestine and can invade the intestinal mucosa or perianal area, causing autoinfection (Sandground, 1925; Streit, 2008; Page *et al*., 2018). In dogs, the infection may be asymptomatic. However, respiratory, dermatologic and gastrointestinal signs accompanied by vomiting and anorexia may occur in young animals, especially in puppies (Robertson and Thompson, 2002; Paradies *et al*., 2017), sometimes with fatal consequences (Dillard *et al*., 2007; Unterköfler *et al*., 2022). So far, none of the available anthelmintics are formally approved for the treatment of strongyloidiasis in dogs. In a few cases, successful off-label treatment has been demonstrated using moxidectin, milbemycin, praziquantel, fenbendazole and ivermectin, often in combination (Eydal and Skírnisson, 2016; Unterköfler *et al*., 2022; Deak *et al*., 2023). However, a single dose is not sufficient to eradicate the parasite, and successful therapy requires repeated applications (Itoh *et al*., 2009; Paradies *et al*., 2017; Basso *et al*., 2019). On the other hand, the administration of some drugs, such as glucocorticoids, can lead to a hyperinfection and worsen the health status (Basso *et al*., 2019), as they immunosuppress the host and act directly on the larvae, promoting accelerated moulting to the autoinfective stage (Ramanathan and Nutman, 2008).

*Strongyloides stercoralis* is being intensively studied in dogs because of its zoonotic potential (Gorgani-Firouzjaee *et al*., 2022). Infected free-roaming dogs could play a role in spreading the infection to other hosts including humans (Jaleta *et al*., 2017; Nagayasu *et al*., 2017) and reciprocally, humans could be a possible source of infection for dogs living in close contact with them, but this is yet to be proven (Bradbury and Streit, 2024). This extremely close coexistence between domestic carnivores and humans has created conditions for the mutual transmission of pathogenic and non-pathogenic organisms and has become part of the One Health concept, especially in Western society (Gray and Young, 2011; van Knapen and Overgaauw, 2014; Wetzels *et al*., 2021).

In the case of *S. stercoralis* two main haplotypes/lineages have been described: the potentially zoonotic haplotype/lineage A, which occurs in humans, dogs and mainly captive NHPs and the likely solely canine haplotype/lineage B (Bradbury *et al*., 2021). While haplotype/lineage A appears to be more widespread in dogs worldwide, haplotype/lineage B has been detected only in Australia, Cambodia, Myanmar and Grenada (Nosková *et al*., 2024). However, numerous cases of *S. stercoralis* infections in dogs are reported without genetic analysis (Schnyder *et al*., 2022), so the exact distribution of individual haplotypes/lineages worldwide is most likely inaccurate.

Basic microscopy, ideally following the Baermann larvoscopy (Schnyder *et al*., 2022), is essential for the detection of first-stage *S. stercoralis* larvae (L1; or occasionally infective L3 and parasitic females) from faecal samples (Basso *et al*., 2019; Schnyder *et al*., 2022), while oval eggs with U-shaped larvae are typical for some other *Strongyloides* species (Viney and Lok, 2015). Genetic-based methods are essential for the resolution of the individual haplotypes/lineages. Both the hypervariable region (HVR-IV) of 18S rDNA and cytochrome c oxidase subunit I (*cox1*) have the discriminatory power to differentiate *S. stercoralis* haplotypes/lineages (Beknazarova *et al*., 2019) and should be selected as the currently most appropriate markers, as there are no other discriminatory methods. Some haplotypes were recognized using the HVR-IV region of 18S, which correspond to the particular *cox1* lineage, indicating that mixed infections with other *Strongyloides* species are not uncommon (Barratt *et al*., 2019) and this could be true also for the haplotypes/lineages of *S*. *stercoralis* in dogs. In recent years, increasingly complex genotyping methods have been used in *Strongyloides* research that enable the detection of multi-haplotypes infections in faecal samples (Barratt *et al*., 2019).

After receiving a sample from a dog with disseminated *Strongyloides* infection, we aimed to uncover which genetic haplotype and lineage is the causative agent of the disease. A high-throughput sequencing (HTS) approach was used to determine whether multiple haplotypes of HVR-IV overlap during this fatal infection. Sanger sequencing complemented the HTS results on the *cox1* and HVR-I regions.

## Material and methods

### Clinical case

A one-year-old crossbred dog female was adopted from a Slovakian shelter in April 2022 with no medical records. From the beginning, the dog had repeated diarrhoea. Before the case was submitted to us, coproscopic examination was either not performed or included only faecal flotation with a negative result. In September 2022, the dog was presented to a veterinary clinic in Hradec Králové, Czech Republic as subfebrile, with profuse diarrhoea, apathy, cachexia (BCS 1/5). A fresh faecal sample was collected and examined using Sheather's flotation at the clinic. Baermann larvoscopy was not performed. Infrequent damaged unspecified larvae were microscopically detected after Sheather's flotation. The hematological examination was unremarkable. In the biochemical profile, liver enzymes and bilirubin were slightly elevated, amylase and pancreatic lipase were markedly elevated together with mild hyponatremia. The dog was dewormed with a single dose of moxidectin combined with imidacloprid (Advocate spot on). Furthermore, amoxicillin/clavulanate (22 mg kg^−1^, Noroclav inject.) and marbofloxacin (3 mg kg^−1^, Marbocyl FD inject.) were administrated. No larvae were detected after 6 days during the control Sheather's flotation. Dog condition did not improve; loss of appetite and vomiting persisted. Radiographic examination of the chest in latero-lateral projection showed a slightly accentuated bronchial pattern; endoscopy revealed abnormal findings in the duodenum, which was markedly hyperaemic throughout and had numerous miliary coagulum. The dog's condition deteriorated, with opisthotonus and bloody, profuse diarrhoea. Therefore, endoscopic examination, biopsy and subsequent histological examination were performed (LABOKLIN GmbH & Co. KG, see Histologic examination below). Based on the histological results, a faecal sample was sent to the University of Veterinary Sciences Brno for coproscopic examination. *Strongyloides* infection was diagnosed based on the microscopic and molecular identification and treated with Flubendazole (Flubenol KH paste) 22 mg kg^−1^ for 3 consecutive days. During the control flotation of the sample from the 3rd day after flubendazole administration (19th day after Advocate spot application), non-motile *Stongyloides* stages were observed. On the fourth day after initiation of anthelmintic therapy, the dog developed severe respiratory distress due to polypnea associated with complete anorexia. The respiratory distress was treated with corticosteroids, methylprednisolone (1 mg kg^−1^, SoluMedrol 40 mg inject.) and opiates (butorphanol 0.2 mg kg^−1^, Butomidor inject.), which led to relief of the polypnea. The dog, however, died the following day.

### Histologic examination

A total of 27 samples, taken during the endoscopy, from the stomach, duodenum, ileum and colon were submitted to the LABOKLIN GmbH and Co. KG, for histological examination. The samples ranged from 1 mm in diameter up to 5 × 2 × 2 mm and included mostly mucosal surfaces. All samples were fixed in 10% phosphate-buffered formalin for 24–48 h to ensure correct fixation. They were then trimmed and processed according to laboratory standards (Fischer *et al*., 2008), embedded in paraffin wax, cut at 4–5 μm thickness and stained with Haematoxylin-Eosin (HE) and Periodic Acid-Schiff stain (PAS). The slides were scanned and analysed using image analysis software (NIS-elements software) (Nikon, Japan); Aperio ImageScope (Leica, Germany).

### Faecal sample preparation, DNA extraction and molecular assays

A 200 ml suspension of faecal sample in water for molecular identification and the same amount of suspension fixed in formalin for coproscopic examination were sent to Department of Pathology and Parasitology, University of Veterinary Sciences Brno, Czech Republic in October 2022. Due to submission of a non-standard sample, the Baermann larvoscopy was not performed. The nematode stages in formalin-preserved sample were let to sediment, collected individually by a pipette and observed and measured using the light microscope with Nomarski differential contrast (Olympus BX41) at 100 × magnification and photographed (Olympus AX70).

The faecal–water suspension was centrifuged in 50-ml falcon tubes. The sediment was transferred into 1.5 ml tubes and dried overnight at 37°C before DNA isolation (Murphy *et al*., 2000). Total DNA from one sample was extracted using the DNeasy PowerSoil Kit (QIAGEN, Germany) according to the manufacturer's protocol. The most frequently used markers for *Strongyloides* spp. amplification were selected (Barratt *et al*., 2019), namely two hypervariable regions (HVRs) of 18S rDNA (HVR-I and HVR-IV) and the mitochondrial gene *cox1*. The HVR-I region (434 bp) was amplified using primers New_HVR-I_F and New_HVR-I_R, whereas *cox1* (650 bp) was amplified with pair primers TJ5207 and TJ5208 (Jaleta *et al*., 2017). The volume and mixture of the PCR reaction of HVR-I region and *cox1* were performed according to Nosková *et al*. (2023) (Table 1). Sanger sequencing was performed by Macrogen Europe (Netherlands). To reveal the potential occurrence of multiple *Strongyloides* haplotypes, the library for HTS was created using a 2-step-PCR approach, performed using primers New_HVR-IV_F and New_HVR-IV_R (Barratt *et al*., 2019), amplifying the HVR-IV region (255 bp). In the first step of PCR inner locus specific primers for the HVR-IV region amplification (New_HVR-IV_F and New_HVR-IV_R) were used, additionally containing nextera adaptor sequences at 5′ ends following design from a 16S Metagenomic Sequencing Library Preparation protocol. The first amplification was performed in a 10 μL reaction volume, including Kapa 2 G Robust Hot Star polymerase (Kapa Biosystems, United States), 0.25 μL of each primer and 2 *μ*L of DNA template, before the second amplification was performed with the same polymerase, 4 μL of Nextera primers with adaptors and 2 μL diluted product in a 20 μL reaction volume (Table 1). The final library was sequenced using MiSeq Reagent Kit v3 (2 × 300 bp pair-end reads) by Illumina MiSeq platform (Illumina, USA).

**Table 1.** Primers and reaction conditions

Primers	Sequence 5′→ 3′	Reaction conditions	Region of gene	References
TJ5207 TJ5208	TTTGATTGTTACCTGCTTCTATTTT TTTTACACCAGTAGGAACAGCAA	2 min at 94°C; 35 cycles of 20 s at 94°C, 15 s at 50°C, 90 s at 72°C; and 7 min at 72°C	*cox1*	Jaleta *et al*. (2017)
HVRI F HVRI R	GCTCATTATAACAGCTATAGACTACACGGTA CCACAACAATCATTTTATGCACTTGG	3 min at 95°C; 35 cycles of 15 s at 95°C, 15 s at 60°C, 15 s at 72°Cs; and 1 min at 72°C	18S rDNA (HVR-I)	Barratt *et al*. (2019)
HVRIV F HVRIV R	CGGGCCGGACACTATAAGG ATCTCTAAACAGGAACATAATGATCACTAC	3 min at 95°C; 30 cycles of 15 s at 95°C, 15 s at 60°C, 15 s at 72°Cs; and 1 min at 72°C	18S rDNA (HVR-IV)	
Nextera adaptor F	TCGTCGGCAGCGTCAGATGTGTATAAGAGACAG	3 min at 95°C; 16 cycles of 15 s at 95°C, 30 s at 55°C, 30 s at 72°C; and 72°C for 3 min	-	16S metagenomic sequencing library preparation protocol, Illumina
Nextera adaptor R	GTCTCGTGGGCTCGGAGATGTGTATAAGAGACAG			

### Data processing and phylogenetic analyses

Raw FASTQ sequences of the HVR-I of 18S rDNA and partial *cox1* genes obtained after Sanger sequencing were checked manually and trimmed in Geneious 9.1.5 (www.geneious.com). Raw HVR-IV of 18S rDNA FASTQ sequences obtained using HTS were trimmed using Skewer (Jiang *et al*., 2014) and denoised using the dada2 package (Callahan *et al*., 2016) in Rstudio (https://www.rstudio.com). The taxonomic identification for all sequences (one from HVR-I, one from HVR-IV and one from *cox1*) were performed using BLAST software against the BLAST nt database (Altschul *et al*., 1990), available online at the National Center for Biotechnology Information, https://www.ncbi.nlm.nih.gov/.

For phylogenetic analyses, 154 sequences of the partial *cox1* gene of *S. stercoralis* from dogs were downloaded from Genbank and checked for duplicates. In addition, 12 sequences from humans and NHPs were included. Phylogenetic analysis of partial *cox1* sequences was reconstructed by Bayesian inference (BI) in MrBayes 3.2.6 (Huelsenbeck and Ronquist, 2001). Only unique dog sequences from 11 localities were included in the phylogenetic analysis. The Muscle alignment (715 bp) of *cox1* consisted of one sequence obtained during the present project and 61 sequences from Genbank, including *Necator americanus* (AJ417719) as an out-group. Nodal support was assessed to 10^6^ replicates. Substitution GTR + G model was used as selected by Modelgenerator (Keane *et al*., 2006).

## Results

### Detection of adult parthenogenetic female and pathological findings

**Figure 1.**
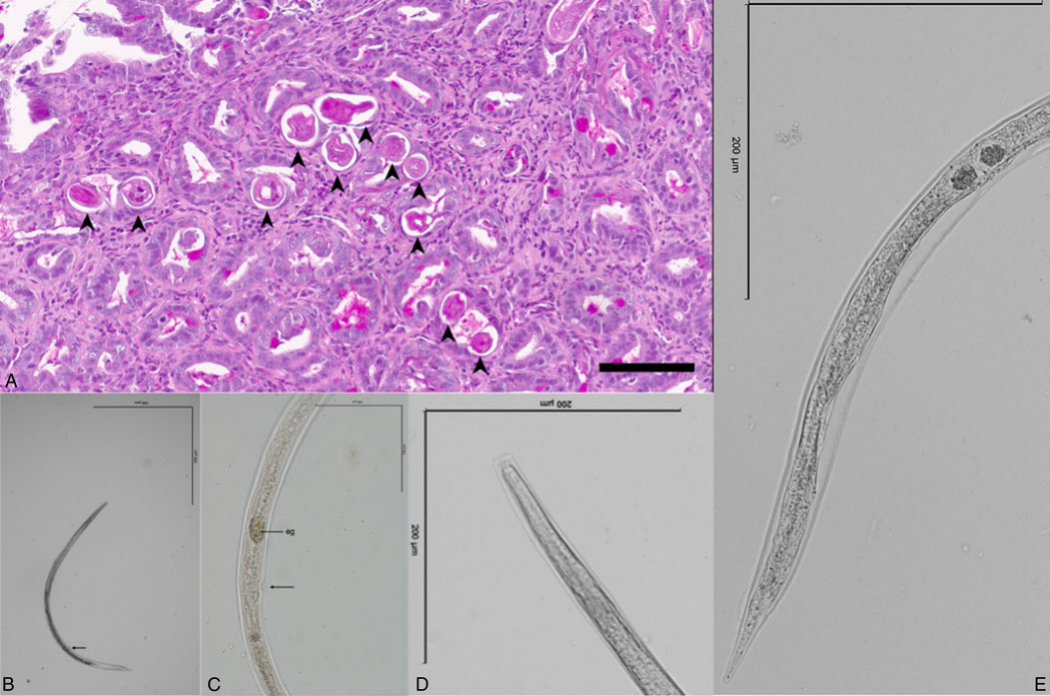
(A) Multiple cross sections of degenerated nematode parasites (arrows) embedded in the intestinal crypt, the remaining mucosal interstitial contains variable numbers of lymphocytes, plasma cells and scattered eosinophils, scale bar 80 μm; (B) general view of *Strongyloides stercoralis* parasitic female from dog, arrow indicates vulva, scale bar 500 μm; (C) vulval region of *S. stercoralis* parasitic female, arrow indicates vulva, scale bar 100 μm, abbreviation: eg – egg; (D) buccal cavity of *S. stercoralis* parasitic female, scale bar 200 μm and (E) tail of *S. stercoralis* parasitic female, scale bar 200 μm.

Within the intestinal samples of duodenum, ileum and colon, the mucosa, lamina propria and submucosa, when available, were diffusely infiltrated and expanded by moderate numbers of lymphocytes, cells, as well as scattered eosinophils. Embedded within the intestinal crypts there were numerous cross and tangential sections of degenerated adult nematodes and larvae (Fig. 1A). The villi were overall slightly blunted and occasionally fused. The intestinal crypts often contained an increased amount of mucinoid material, as well as a small number of cellular debris. The associated lymphoid tissue in these areas was often prominent and hyperplastic. The stomach samples were histologically unremarkable, with no evidence of parasites or overt inflammation, other than mild superficial erosion and apical haemorrhages. High number of adult parthenogenetic *S*. *stercoralis* females (average length 1300 μm) were observed in faecal suspension using microscopy. The typical morphological characters such as elongated (filariform) oesophagus (Fig. 1B), vulval region (Fig. 1C), buccal cavity (Fig. 1D) and tail (Fig. 1E) were observed.

### Haplotypes and phylogenetic analysis of *S. stercoralis*

**Figure 2.**
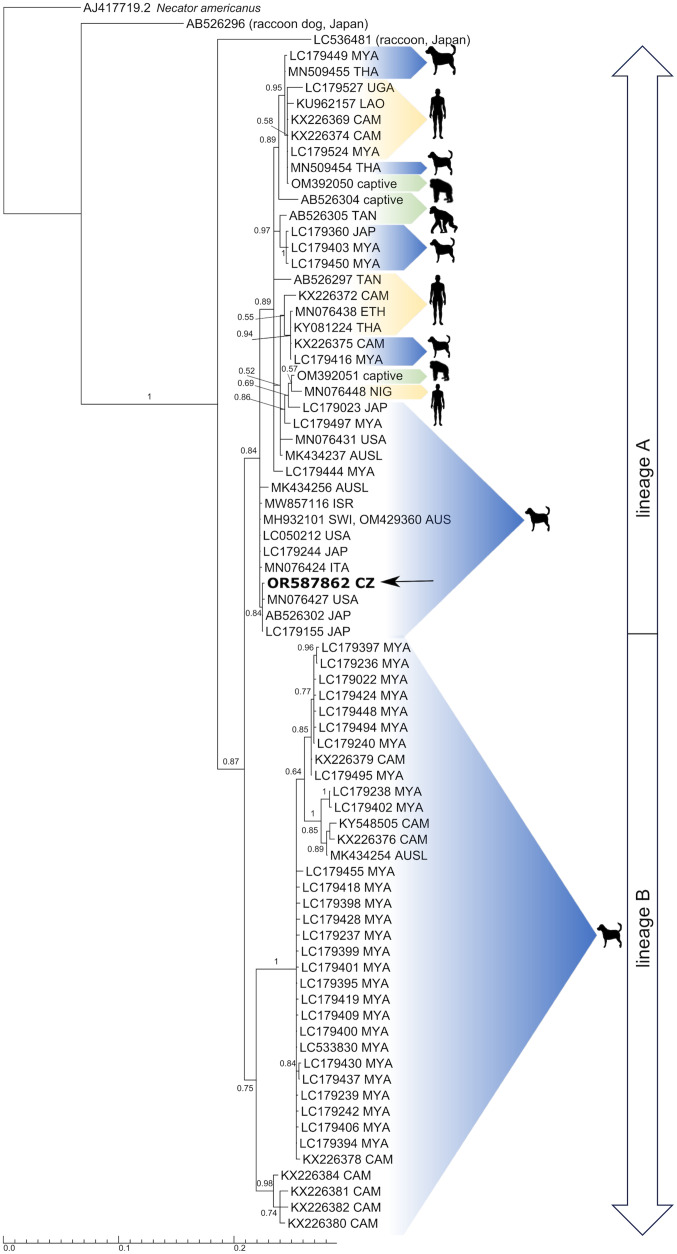
Bayesian inference phylogenetic tree inferred from cox1 (715 bp) calculated from a muscle-constructed alignment using GTR + G model for nucleotide substitutions. Numbers at the branches indicate Bayesian posterior probability based on 10^6^ replicates. Branch lengths indicate expected numbers of substitutions per nucleotide site. Sequence obtained during the current study is in bold.

*Strongyloides stercoralis* sequences of the HVR-I (466 bp) and HVR-IV (290 bp) of 18S rDNA and partial *cox1* (628 bp) were obtained from the examined dog faecal sample. BLAST search showed that sequence of the HVR-I region corresponded with haplotype VI while HVR-IV corresponded with haplotype A as marked in Barratt *et al*. (2019). Moreover, 97 046 high-quality reads were obtained using HTS and only potentially zoonotic HVR-IV haplotype A was detected, while the solely canine haplotype B was not recorded. The *cox1* results also detected the potentially zoonotic lineage A. The resulting BI tree based on *cox1* showed two separate *S*. *stercoralis* clades corresponding to lineages A and B, with our *cox1* sequence (OR587862) placed in the A lineage (Fig. 2), clustering with high support (0.84 BI) into a sub-clade comprising two isolates from Japanese dogs and one isolate from a dog from USA. The pairwise sequence distance (PSD) within this sub-clade did not reach over 0.2% but differ from other sub-clades within lineage A from 1.8% to 4.2%. Two distinct subclades are shown also within lineage B and differed by 4 to 6.2%. Clades containing lineage A and B do not differ by more than 8.3%.

## Discussion

Surveillance of *S*. *stercoralis* in dogs, including molecular identification of positive cases, should be an important part of health management that can lead to a reduction in the risk of the parasite's impact on host health, as well as a reduction in fatal cases. Knowledge of particular *S. stercoralis* haplotypes/lineages helps evaluate the risk of zoonotic transmission to humans (Nagayasu *et al*., 2017). However, the combination of coproscopic and genetic analyses is not routinely performed in dogs. Here we present a case report of disseminated *S*. *stercoralis* infection in a dog evaluated by a combination of histology, microscopy and HTS approaches, with an appeal for the correct treatment approach.

Infections of dogs with *S. stercoralis* are considered rare in the Czech Republic. Only one case has been documented in the literature, reporting unidentified *Strongyloides* eggs found in dog faeces (Valkounová, 1982). Since only *S. stercoralis* L1 are typically detected in faeces of infected dogs, evaluation of this case is speculative. Recently, 9-week-old Chihuahuas imported to Switzerland from a Czech breeding station, were tested positive for *S. stercoralis*, suggesting that the infection had already been introduced from the Czech Republic (Schnyder *et al*., 2022). Overall, cases of canine strongyloidiasis have emerged throughout Europe in the last decade (e.g. Dillard *et al*., 2007; Riggio *et al*., 2013; Eydal and Skírnisson, 2016; Paradies *et al*., 2017; Bourgoin *et al*., 2018; Basso *et al*., 2019; Hall *et al*., 2020; Schnyder *et al*., 2022; Unterköfler *et al*., 2022; Deak *et al*., 2023) suggesting an increasing occurrence or recent spread across Europe, likely due to the importation of dogs (Schnyder *et al*., 2022), including from *Strongyloides*-endemic countries (Unterköfler *et al*., 2022). However, improved awareness, diagnosis and immunosuppression in dogs may also play a role in the increased number of cases (Cervone *et al*., 2016). The dog in this study was adopted from Slovakia, which is considered one of the endemic countries for *Strongyloides* in both dogs and humans (Štrkolcová *et al*., 2017). In addition, transmission of *S. stercoralis* between humans and dogs was suspected as IgG antibodies against *S. stercoralis* were detected in serum samples from children and rhabditiform larvae of *S. stercoralis* were detected in dogs in a Roma settlement in Slovakia (Štrkolcová *et al*., 2017), although without molecular data. Further reports of strongyloidiasis in humans, but without molecular data, have been reported mainly from Eastern and Southern Europe (Ottino *et al*., 2020), possibly influenced by increasing global migration (Asundi *et al*., 2019; Marrone *et al*., 2023).

Although most case reports and prevalence studies of infected dogs with *S. stercoralis* were based on microscopic diagnostics, some studies also included the genotyping of *Strongyloides* (Basso *et al*., 2019; Salant *et al*., 2021; Unterköfler *et al*., 2022). Unfortunately, the haplotypes/lineages are not always reported, as in the case of Chihuahuas imported into Switzerland from the Czech Republic (Schnyder *et al*., 2022). So far, only haplotype/lineage A has been detected in dogs in Europe, including the presented case (Nosková *et al*., 2024). In the Czech Republic, the potentially zoonotic *S. stercoralis* haplotype A was also molecularly confirmed in captive orangutans kept in zoological gardens (Nosková *et al*., 2023). In humans in Europe, haplotype A has only been confirmed in Italy (Barratt *et al*., 2019).

Until now, studies on *Strongyloides* infection in dogs carried out in Europe have been conducted based on classical sequencing, which has its limitations when it comes to detecting multiple haplotypes. Conventional PCR analyses followed by Sanger sequencing provide only a single sequence and are therefore suitable for detection of *Strongyloides* from a single larva, for example, but the molecular identification of *Strongyloides* from total DNA extracted from faeces, which may contain multiple *Strongyloides* haplotypes, requires more complex genotyping approaches (Nosková *et al*., 2024). Hypothetically, the almost complete absence of haplotype B could be due to the dominance of haplotype A in potentially mixed infections, which is preferentially detected by Sanger sequencing. Though, only the potentially zoonotic haplotype A was detected in our study when HTS was applied, and the previous fatal cases of disseminated strongyloidiasis analysed by molecular tools also revealed only the potentially zoonotic haplotype A so far. (Basso *et al*., 2019; Unterköfler *et al*., 2022). It appears that haplotype A is pathogenic or at least pathogenic under certain conditions, while the pathogenicity of haplotype B infecting dogs is so far unknown. However, in many studies, the haplotype was not identified, and it should be considered in the future. Perhaps if veterinary clinics, together with specialized institutions (e.g. universities, genomic or research centres), have the opportunity to perform HTS identification, the valuable results would fill the gaps regarding the genetic diversity and distribution of *Strongyloides* infection and clarify the possible pathogenicity of haplotype B.

In general, *S. stercoralis* disease progression in dogs varies from a latent course, especially in adult immunocompetent dogs, to fatal cases in young or immunocompromised dogs (Cervone *et al*., 2016). Clinical signs include diarrhoea, erythema, respiratory problems, epaxial atrophy, nervous signs such as paresis, tremor, comatose states, recurrent epileptiform seizures, torticollis (Dillard *et al*., 2007; Basso *et al*., 2019; Schnyder *et al*., 2022; Unterköfler *et al*., 2022) or opisthotonus as in our study. Respiratory symptoms are attributed with *Strongyloides* infection as L3 enter the bloodstream and migrate through the trachea into the intestine (Page *et al*., 2018). Disseminated strongyloidiasis develops when the host is exposed to long-term autoinfection. *Strongyloides* larvae have previously been found post-mortem not only in the intestinal mucosa and respiratory tract, but also in the CNS, spleen, kidneys and muscles (Thamsborg *et al*., 2016; Paradies *et al*., 2017). Strongyloidiasis in humans has a similar course. Most infections are mild, but under certain conditions (e.g. if the patient is immunosuppressed) dissemination may occur. While the mild course of the disease is characterised by gastrointestinal, pulmonary and dermatological symptoms, the infection can lead to severe systemic disease with fatal consequences. In a disseminated form, the larvae can occur in any organ and lead to systemic bacterial sepsis (Hagelskjaer, 1994; Qu *et al*., 2016; Karanam *et al*., 2021). In the case of the dog described in this study, the necropsy was not authorised by the dog's owner. However, we detected typical pathologies in the duodenum, ileum and colon by endoscopy. In addition, we observed parasitic females in the faecal sample, which is rare and suggests disseminated disease (Basso *et al*., 2019).

The treatment of strongyloidiasis has not yet been clarified, as efficacy has only been surveyed in a small number of dogs. Ivermectin and fenbendazole appears to be the most effective anthelminthics, although *S*. *stercoralis* infection in dogs is not listed in indications of any canine anthelminthics containing these substances (Paradies *et al*., 2017; Schnyder *et al*., 2022). Nevertheless, a single administration is not considered sufficient, as the excretion of the larvae in the faeces persists (e.g. Paradies *et al*., 2017; Schnyder *et al*., 2022) and a repetition of the therapy at different intervals is usually necessary (Thamsborg *et al*., 2016). Single dose of ivermectin is particularly insufficient for disseminated infection (Buonfrate *et al*., 2022). Although the examined dog was treated against intestinal parasites, the effect on *Strongyloides* infection was insufficient, although the dog was classified as negative after the first treatment. Control of *S. stercoralis* infection can only be considered successful if the faecal examination repeatedly shows a negative larvoscopy result (Schnyder *et al*., 2022). Unfortunately, in most cases of disseminated strongyloidiasis, prolonged therapy is not possible due to the deteriorating condition of the patient (Basso *et al*., 2019). Patients with the disseminated form often suffer from secondary bacterial infections, respiratory failure, requiring appropriate intensive care (Cervone *et al*., 2016). The development of a disseminated form can be promoted by the use of glucocorticoids (Mansfield *et al*., 1996). Even short courses (6–17 days) of corticosteroids in immunocompetent patients without underlying immunosuppressive conditions have even been associated with death (Krolewiecki and Nutman, 2019). If a *Strongyloides* infection is detected in a dog taking corticosteroids, their use must be ceased immediately as there is a risk of development of the disseminated form (Graham *et al*., 2019). The fatal case of the disseminated form in the examined dog was probably triggered by the prescription of corticosteroids. However, the disseminated infection in the studied dog developed before the administration of corticosteroids, when the dog showed opisthotomus, as the central nervous system can also be affected in a disseminated infection (Concha *et al*., 2005).

## Conclusion

The increasing occurrence of *S*. *stercoralis* in dogs in various European countries shows that this parasite appears to pose an increasing risk in Europe. The cross-border transportation of dogs from endemic countries and the accumulation of dogs in animal shelters could contribute to the spread of the infection. So far, only the potentially zoonotic haplotype/lineage A is known to be circulating in dogs in Europe, which in turn could pose a risk to human health. Due to the potential for cross-transmission between humans and dogs, *S. stercoralis* is a classic candidate for One Health approaches, where attention must be paid to accurate control and diagnosis in both humans and dogs. The correct treatment approaches should include: (1) Appropriate methods for the detection of *Strongyloides* infection, such as the Baermann larvoscopy or PCR, should be included in routine diagnostics in dogs. If faecal flotation is the only diagnostic method, the diagnostic reports should clearly state that the method performed cannot reliably detect *Strongyloides* so the veterinarians who commission the test are aware. (2) The repeated therapy and the confirmation of the treatment success should be stated only after several negative larvoscopy results. (3) If the *Strongyloides* infection is detected in a dog taking corticosteroids, their use must be ceased immediately as there is a risk of development of disseminated form. (4) Subsequently, appropriate molecular tools, ideally using HTS approaches, are highly recommended for *Strongyloides*-positive samples to raise awareness of the spread of individual *S. stercoralis* haplotypes/lineages. (5) General awareness of this spreading parasite should be raised among physicians, veterinarians and clinicians, together with caution in the use of glucocorticoids.

## Data Availability

Sequence based *cox1* of *Strongyloides stercoralis* is available and uploaded to GenBank under the accession numbers OR587862.
